# Onychomycosis: Potential of Nail Lacquers in Transungual Delivery of Antifungals

**DOI:** 10.1155/2016/1387936

**Published:** 2016-03-30

**Authors:** Nida Akhtar, Hemlata Sharma, Kamla Pathak

**Affiliations:** ^1^Department of Pharmaceutics, Rajiv Academy for Pharmacy, P.O. Chhatikara, Mathura, Uttar Pradesh 281001, India; ^2^Department of Pharmaceutics, Pharmacy College Saifai, Saifai, Etawah, Uttar Pradesh 206130, India

## Abstract

Onychomycosis constitutes the most common fungal infection of the nail (skin beneath the nail bed) that affects the finger as well as toe nails. It is an infection that is initiated by yeasts, dermatophytes, and nondermatophyte molds. Nail lacquers are topical solutions intended only for use on fingernails as well as toenails and have been found to be useful in the treatment of onychomycosis. Thus, in the present review an attempt has been made to focus on the treatment aspects of onychomycosis and the ungual delivery of antifungals via nail lacquer. Several patents issued on nail lacquer till date have also been discussed. Penetration efficiency was assessed by several researchers across the human nail plate to investigate the potentiality of nail lacquer based formulations. Various clinical trials have also been conducted in order to evaluate the safety and efficacy of nail lacquers in delivering antifungal agents. Thus, it can be concluded that nail lacquer based preparations are efficacious and stable formulations. These possess tremendous potential for clinical topical application to the nail bed in the treatment of onychomycosis.

## 1. Introduction

Onychomycosis is described as a fungal infection affecting primarily finger and toe nails. It is reported to influence about 19% of the population across the world and constitute about 50% of all the nail diseases [1]. It is sometimes thought to be a trivial disorder acquiring only cosmetic implications and might lead to embarrassment underlining self-esteem. If it is not treated well it can lead to discomfort and may spread to the nearby tissues. It is a frequently occurring disease of nails which is initiated by nondermatophyte molds and dermatophytes. Nondermatophyte filamentous fungi are considered as a causing agent of onychomycosis. The prevalence of this disease may vary across the globe because of several cultural and socioeconomic factors. Prevalence of the infection also depends upon the geographic area, population, and mycological features or diagnostic methods employed [2]. Onychomycosis is considered as one of the common dermatology based conditions. Enhanced survey on the prevalence of the disease as well as inventions of newer antifungal agents resulted in higher concern among the patients to get cure of the disease and also among medical practitioners to constitute an effective therapy. However, there has been an issue regarding the treatment which may be prescribed without prior knowledge of infection. The effectiveness of several antifungals against the fungi is not well understood and drugs are often recommended for inappropriate time periods [3]. Therefore, the treatment of onychomycosis is a challenging task as the infection is embedded within the nail. It may take a year or more to get cure as new nail growth must completely replace the old and infected one [4]. Imidazole derivatives among several antifungals are reported to be effective in the management of onychomycosis. These are broad spectrum antifungal agents and are active against mold, yeast, dermatophytes, and some bacteria of the actinomycetes and highly active against some Gram-positive cocci and bacilli. Antifungals have been available in market in the form of cream, ointment, lotion, powder, and solutions. These formulations require high concentration of active agents to be incorporated for effective therapy because of their low efficacy. To overcome the limitations of conventional formulations there is a need of an effective system that can deliver the antifungals deep into the nail bed. However, the development of a formulation for safe and effective topical delivery in the treatment of onychomycosis is still under infancy.

Topical therapy would be an attractive alternative approach in the treatment of onychomycosis as it is found to be capable of overcoming most of the limitations of systemic administration and targeting the drug at its site of action, with minimum interactions and adverse effects. Limited permeability of the drug through nail plate and blood supply in the affected area might lead to subtherapeutic concentration and can be overcome via topical application [5]. Further, most of the conventionally used formulations are not specifically adapted to the nail since they are readily removed by rubbing or washing [6]. To overcome these barriers, ungual delivery can be opted for the delivery of drug across the nail plate. Medicated nail lacquer formulations have been developed for effective ungual drug delivery of antifungals to improvise the treatment of onychomycosis. In one of our in-house research reports, potential of nail lacquer has been investigated for the treatment of psoriasis. A safe, stable, and efficacious nail lacquer of isotretinoin was formulated for effective topical clinical application. Drug targeting has been done into the nail bed in order to treat nail psoriasis [7]. So the development and evaluation of nail lacquer for its therapeutic efficacy are a well-established concept. Thus, in the present review an attempt has been made to focus on the treatment aspects of onychomycosis and the delivery of antifungals via nail lacquer for ungual delivery. Several clinical and patent reports have also been reviewed.

## 2. Onychomycosis and Its Etiology

Onychomycosis is defined as a fungal infection affecting nails of finger or toes resulting in thickening, discoloration, and separation from the nail bed. Onychomycosis affects about 10% of the common population but is more prevalent in adults. The risk factor of onychomycosis is 1.9-fold to 2.8-fold more in persons suffering from diabetes compared to the common population. However, the prevalence varies from 15 to 40% in case of patients infected with human immunodeficiency virus [4]. Onychomycosis is classified into two types. The first type is distal subungual onychomycosis affecting nail plate, nail bed, and hyponychium (infection more prominently spreads from tinea pedis from the surrounding skin). The diseased nails become dystrophic and thick. The color of nails modifies from yellowish-white to brown. The second type is proximal subungual onychomycosis in which the fungus usually spreads through the proximal nail folds. Clinically, the nail has a whitish appearance. A clinical type, endonyx form of onychomycosis, affecting the nail plate without onycholysis or hyperkeratosis is also known. Superficial white type of onychomycosis (nail becomes white with chalky texture) is observed to be less frequent. The fungus causing this includes* T. mentagrophytes*, but nondermatophyte fungi (*Aspergillus*,* Fusarium*, and others) can also produce these changes [8, 9].

## 3. Transungual Drug Delivery

Transungual drug delivery is defined as system which is related to drug transport across the nail to gain targeted drug delivery in order to treat nail diseases. In the term transungual, “Trans” signifies “through” and “unguis” signifies “nails” [10]. Transungual drug transport system is thought to be quite effective to manage nail disorders because of its better adherence, localized action, which provides minimum systemic side effects [11]. Ungual therapy provides several benefits over oral/systemic drug delivery such that preparation is easy compared to oral dosage forms like tablets, and so forth. Drug interactions and systemic adverse effects are absent. Less common local rash related side effects like periungual erythema of the proximal nail fold gradually disappear after a few minutes and usually get less over time as the body adjusts to the new medication. Systemic absorption is less and it is based on application of topical formulation that can be easily removed when needed. Ungual therapy provides improved adherence and is suitable for those who are unable to take systemic medication. It is preferable in case of elder patients and patients receiving multiple medications so as to avoid drug-drug interactions. In spite of possessing several benefits, it also poses various problems like composition of the nail plate, limits penetration of drugs, and allows only a fraction of topical drug to penetrate across and hence the desired therapeutic concentration is not achieved [11].

## 4. Approaches for Transungual Drug Delivery

**Figure 1:**
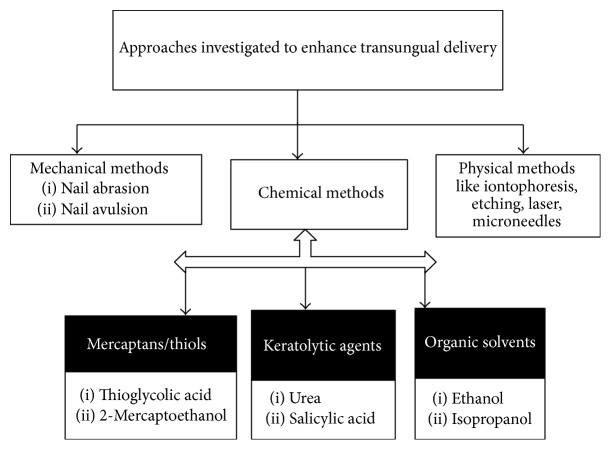
Various approaches for effective transungual drug delivery.

Nail penetration can be achieved by various methods such as mechanical, chemical (permeation enhancers), and physical methods (like iontophoresis, microneedles, etc.) as discussed in the preceding text and highlighted in Figure 1.

### 4.1. Mechanical Methods

Mechanical methods such as nail abrasion and avulsion have been investigated by several researchers. Nail abrasion is methodology which causes thinning of the nail plate, reducing the fungal debris in case of onychomycosis. Nail abrasion describes sanding of the nail plate in order to minimize its thickness or damage it in a complete form. Sand paper (# 150 or 180) may be used on the basis of intensity. Sanding should be carried out on the edges of nails. A high-speed sanding hand piece can be utilized for this purpose. Further, dentist's drills have also been used to create small size holes in the nail plate, thus improving the penetration [42].

On the other hand, nail avulsion either completely or partially is the removal of whole nail plate surgically or removal of infected nail plate carried out under the action of local anesthesia. Keratolytic substances like urea or salicylic acid may cause softening of the nail plate for avulsion [12].

### 4.2. Chemical Methods

A combination of 2-mercaptoethanol and N-acetyl-l-cysteine is depicted to improvise the delivery of tolnaftate, into the nails. The penetration of oxiconazole via N-acetyl-l-cysteine has also been documented [43]. Keratolytic agents like papain, urea, and salicylic acid were used in enhancing the penetration of few antifungals like ketoconazole, miconazole, and itraconazole [44]. Furthermore, organic solvents like ethanol, isopropanol, propylene glycol, and polyethylene glycol can be employed in enhancing the drug penetration across the nail. Direct interaction of organic solvents present in formulation with the nail plate in case of transungual drug transport might result in enhanced barrier resistivity of the nail. Secondly, use of organic solvent may manipulate the hydration state of nail [45].

### 4.3. Physical Methods

Microneedles based drug delivery systems include the use of arrays of microscopic size needles. These open the pores present in stratum corneum. Further, these also possess the benefit of being so short that they do not stimulate the pain fibers [46]. Another approach is the use of etching technique. Etching occurs via exposure of surface-modifying chemical (e.g., phosphoric acid). It causes the formation of profuse microsporocytes, can enhance the wettability along with surface area, and can decrease the contact angle. An optimum surface is provided for bonding material. Additionally presence of microporosities improves interpenetration and bonding of a polymeric delivery system and facilitation of interdiffusion of a therapeutic agent [47].

Iontophoresis involves the application of electric field for the delivery of a compound across a membrane. Drug diffusion through the hydrated keratin of a nail may be enhanced by iontophoresis. Iontophoresis significantly enhanced drug penetration through the nail. The delivery of griseofulvin was enhanced by 8-fold with the help of iontophoresis [48]. Another significant technique uses carbon dioxide laser. CO_2_ laser methodology might have produced effective but uncertain outcomes. Two techniques have been reported up till now. One is based on the avulsion of the nail part accompanied by treatment with laser at 5000 W/cm^2^. Thus, via this method deep tissue is directly exposed to laser treatment. Second methodology includes penetrating across the nail plate by laser beam of CO_2_ [49].

An emerging approach is based on hydration and occlusion. Hydration enhances the pore size of nail matrix, influencing the ungual penetration. Hydrated nails are found to be more permeable and elastic. Studies based on iontophoresis use this characteristic in order to further improvise the penetration. The pH of the solution and ionic strength have revealed no remarkable influence on nail hydration. Water and drugs diffusivity is enhanced when human skin becomes more hydrated [50]. Electroporation of an advanced physical technique is carried out with the use of an electric pulse of approximately 100–1000 V/cm. This causes disturbance in the lipid bilayers and formation of transient aqueous pores, thus allowing the solutes to pass through nail [46].

## 5. Nail Lacquers for Transungual Drug Delivery

Nail lacquers have been employed as a cosmetic in order to provide protection to the nails and to decorate the nails. Medicated nail lacquers constitute an innovative type of formulations that have been used for transungual drug delivery.  Table 1 reviews several market formulations based on nail lacquers. These preparations consist of a solution of a film-forming polymer and drug [12].

**Figure 2:**
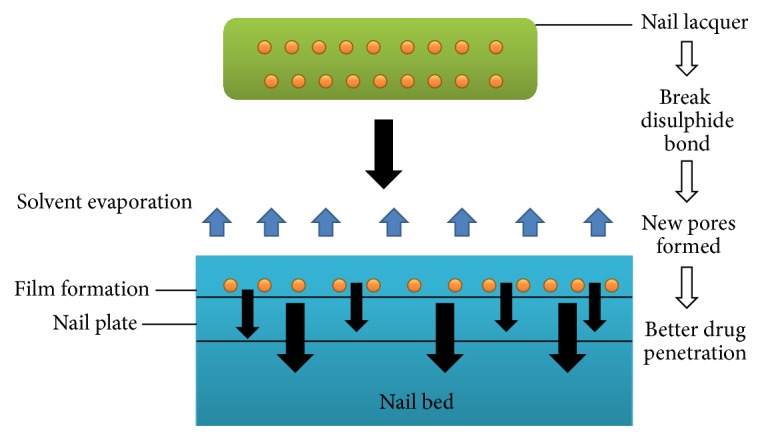
Mechanism of drug penetration via nail lacquers.

Upon application to the nail plate, solvent evaporation takes place. The film left behind after solvent evaporation works like a drug depot. From this drug store, drug undergoes release and penetration across the nail for an optimum time period. High diffusion gradient is generated for drug permeation into the nail plate [49]. Film formation on nail plate also causes reduction in water loss from the surface of nail surface into the atmosphere. Hyperhydration of the upper nail plate layers takes place, further assisting in drug diffusion. The active agent penetration can further be improvised via use of penetration enhancers like thiol compounds, hydrating agents, and keratolytic agents [51].  Figure 2 highlights the mechanism of drug via nail lacquer.

## 6. Delivery of Antifungal Drugs: Treatment of Onychomycosis

Antifungal drugs have been widely used to treat topical infections of nails.  Table 2 lists various antifungals that have been used in the treatment of onychomycosis. As the occurrence levels of onychomycosis keep on increasing nowadays, need of topical therapy that can circumvent the threats posed by systemic delivery is gaining prominent deliberations.

Various researchers across the globe have worked in the development of effective carriers for antifungal agents.  Table 3 highlights the delivery antifungals via other carriers to treat onychomycosis.

In the preceding text various preclinical/clinical reports are cited that depict the safety and efficacy of nail lacquers in delivering antifungal drugs for the treatment of onychomycosis.

Kushwaha et al. had investigated the potential of AR-12 (antifungal drug) to penetrate into and across the human nail plate.* In vitro* permeation studies were performed across the human cadaver nail plates using Franz diffusion cells. The cumulative amount of drug permeated across the nail plate for one week was found to be 0.82 ± 0.11 ng/cm^2^. The amount of drug retained in active diffusion area of the nail plate was observed to be ~0.42 ± 0.02 *μ*g/mg. TranScreen-N clearly showed that PEG 400 could be a potential enhancer of transungual delivery of AR-12. Further,* in vitro* permeation studies conducted along with 10% PEG 400 showed 6-fold enhancement in transungual drug delivery in comparison to the control solution. The drug loaded in the nail plate was depicted to be 2-fold higher in presence of 10% PEG 400 [52].

In a report by Naumann et al. four systems, namely, nail lacquers, colloidal carriers, solution, and hydrogel of an antifungal drug (EV-086K), were formulated and compared for their efficacy.* Ex vivo* penetration studies using human nails and bovine/equine hoof membranes reported best diffusion rate by the nail lacquer and colloidal carriers (55% and 45% of the applied drug after 24 h, resp.). It was observed that, after 3 h, colloidal carriers could transport the drug to a higher extent. Real time penetration investigation was also conducted by Teflon online diffusion cell in combination with FTIR-ATR spectroscopy. It was depicted that nail lacquer acquired a drug content of about 12% because of the drying that occurred in donor region of the online diffusion cell after ethanol (58.6%) underwent evaporation partially. Though the solution showed maximum permeability coefficient initially, it could exhibit higher drug concentration of only 35% in the acceptor cell after 24 h. On the other hand hydrogel depicted minimum penetration rate and the most marked lag time of about 3 h. The experimental results were found to be entirely different in case of bovine hoof membranes as compared to the equine hoof membranes. EV-086K concentrations in contrast to the applied dose demonstrated conflicting outcomes in contrast to that obtained with using equine hooves. Solution was found to conquer the barrier up to a greater extent (65% in 24 h). In case of colloidal carrier and nail lacquer, 35–40% of the applied drug concentration was determined in the acceptor cell. With application of hydrogel, less drug concentration was detected in the acceptor compartment. Colloidal carriers and solution revealed higher permeability coefficients than hydrogel and nail lacquer in linear range. Further, colloidal carriers showed small lag time as compared to the other formulations which were found to be beneficial for ungual delivery. Combining the results of the studies faster drug penetration rate was concluded via colloidal carriers than nail lacquer [53].

In addition to novel antifungals, the established antifungals have also been explored for transungual delivery. Vipin et al. developed medicated nail lacquer loaded with miconazole with the aim to provide sustained drug delivery. Nail lacquer based formulations were prepared by simple mixing. Among all the formulations, nail lacquer containing drug (2%), nitrocellulose (3%), ethyl cellulose (0.5%), salicylic acid (20%), propylene glycol (5%), and urea (5%) in hydrogen peroxide showed better gloss, nonvolatile content, smoothness to flow, drug content, drug release, and antifungal activity. Diffusion studies conducted across artificial membranes using Franz diffusion cell showed drug release of about 96.19% in 20 h. The permeation characteristics and hydration by propylene glycol improvised the drug permeation. To sustain drug release ethyl cellulose was taken at concentration of 0.25% and the findings depicted extended release of 94.25% up to 36 h. Further, sustained drug release was obtained by increasing the concentration of ethyl cellulose up to 0.5%, which can sustain the drug release up to 48 h. A cumulative drug release of about 96.03% (48 h) was depicted. Further,* in vitro* ungual permeation studies were carried out using bovine hoof membrane. Optimized formulation exhibited optimum rate of diffusion across the membrane and hence was selected for* in vitro* ungual permeation studies. The findings demonstrated superimposable diffusion profiles. This confirmed that artificial cellophane membrane can impersonate the features of* ex vivo* bovine hoof membrane. The authors concluded that miconazole nail lacquer possessed effectiveness in inhibiting the growth of the nail fungi,* Candida albicans.* Therefore, nail lacquer based formulation loaded with miconazole can be employed for transungual drug delivery for management of onychomycosis [54].

On similar lines Dessai et al. formulated nail lacquer for preungual delivery of fluconazole for the management of onychomycosis. Diffusion studies across artificial membrane showed that the formulation containing minimum polymeric concentration and maximum concentration of penetration enhancers showed highest cumulative percent drug release (95.55%). Conversely, the formulation prepared with maximum polymeric concentration and minimum concentration of penetration enhancers exhibited the most sustained drug release (85.90%, 10 h).* In vitro* transungual permeability investigations using cattle hooves obtained depicted drug release of about 99.52% (in 22 h). From both* in vitro* diffusion and* in vitro* permeation investigations, it was depicted that thioglycolic acid could work as an effective penetration enhancer in contrast to dimethyl sulfoxide. This enhancing action of thioglycolic acid was attributed to its small molecular weight and its damaging effect on keratin structure. Reduced lipid content present in the nail layer (dorsal) caused by thioglycolic acid loosened the nail structure, thus facilitating fluconazole to undergo penetration easily. Further, antifungal activity was evaluated by cup plate method against* Candida albicans*. Inhibition zone in case of pure drug was observed to be 26 mm and for optimized formulation it was 25.4 mm. Thus, it was concluded by the authors that optimized formulation was as efficient as the pure drug [55].

The effect of permeation enhancer and molecular weight on the permeability of several antifungal drugs was also established by Miron et al. Formulations containing geraniol, penetration enhancer, antioxidant, and ethanol were prepared. Lipo-lacquer was then prepared by taking lacquer base, geraniol, and ethanol. Permeation studies were conducted across bovine hoof membranes for all the formulations. Further, formulations containing geraniol, nerol, fluconazole, miconazole, fluconazole butenafine hydrochloride, and terbinafine hydrochloride were prepared and subjected to permeation studies. From the results, it was observed that ascorbic acid (used as an antioxidant) can significantly improve the geraniol permeability across the membrane in comparison to the control formulation. Ascorbic acid acts by modifying the structure of keratin, thus improving the permeability. Further, presence of acetylcysteine in the formulations improvised the geraniol permeability. Relation between permeability and molecular weight of ionized drugs (miconazole, fluconazole, terbinafine, and butenafine hydrochloride) and unionized drugs (geraniol, nerol) was also established. The results concluded that molecular weight of drug significantly affects drug permeability as compared to the degree of dissociation. Permeability coefficients of geraniol and nerol were 30- and 175-fold higher than terbinafine and miconazole, respectively. Differences were observed because of their differences in molecular weight. Thus, it was concluded by the authors that an inverse relationship existed between logarithm of permeability and molecular weight [56].

The use of hydroxypropyl-*β*-cyclodextrin (HP-*β*-CD) was studied as an effective transungual permeation enhancer for terbinafine hydrochloride delivery by Chouhan and Saini. A nail lacquer based formulation was developed with an aim to transport the drug across the nail.* In vitro* transungual permeability studies were carried out using nail clippings placed in Franz diffusion cell. From the findings, it was observed that the formulations prepared with HP-*β*-CD exhibited greater flux than the control formulation. The formulation having HP-*β*-CD (10% w/v) depicted maximum flux of 4.586 ± 0.08 *μ*g/mL/cm in comparison to the control formulation having flux 0.868 ± 0.06 *μ*g/mL/cm. This investigation suggested the use of HP-*β*-CD as an effective permeation enhancer for transungual delivery of terbinafine hydrochloride [57].

Hafeez et al. formulated ketoconazole loaded nail lacquer for transungual delivery. In this investigation, the efficacy of nail lacquer in improvising penetration of [14C]-ketoconazole was evaluated by comparing nail distribution, absorption, and penetration. Penetration efficacy was then compared with ketoconazole cream.* In vitro* investigation was carried out by selecting finite dose model. To the human nail plates, all formulations were applied up to seven days. The absorption of drug was determined by observing appearance rate in each nail layer and in the supporting bed. After completion of the study, cumulative amount of drug in the deeper portion of nail layer and nail bed was found to be notably higher than marketed form of ketoconazole (*p* < 0.05). These findings suggested that the delivery of ketoconazole via nail lacquer possessed tremendous potential to treat onychomycosis [58].

Another report on ketoconazole by Shireesh et al. claims ungual delivery of ketoconazole via nail lacquer for treatment of onychomycosis.* In vitro* permeability studies were conducted across the human nail plates employing Franz diffusion cells which reported cumulative drug permeation of 92.10 ± 0.08 and 84 ± 0.04 in case of thioglycolic acid and urea hydrogen peroxide, respectively, at the end of 24 h. Significantly greater permeation was observed in case of thioglycolic acid in comparison to the urea peroxide. These findings claim thioglycolic acid to be a better permeation enhancer than urea hydrogen peroxide in improving the permeability. Thus, it was concluded by the authors that nail lacquer prepared by taking thioglycolic acid can prove to be remarkably better in treating onychomycosis by delivering ketoconazole deep into the nail bed [59].

Traynor et al. demonstrated the influence of permeation enhancers on two marketed nail lacquers and terbinafine transport across the human nail samples* in vitro* using modified Franz cell. It was observed that permeation enhancing system enhanced the permeation of both drugs present in nail lacquers and terbinafine through human nails. Further, ATP assay assured that the system also improvised the permeation of terbinafine across infected cadaver nail, thus causing decreased levels of ATP equivalent to those of uninfected negative control samples. It was concluded that the addition of permeation enhancers modifies the chemical structure of nail. These enhancers not only improvised the therapeutic efficacy of the existing topical products but also initiate transungual transport and efficacy of terbinafine [60]. Gunt and Kasting evaluated the influence of hydration on ketoconazole penetration across the excised human nails. Nails processed with [3H]-ketoconazole solvent kept on the dorsal surface, maintained at 32°C in incubators and exposing them sequentially to relative humidity of 15, 40, 80, and 100% for a period of about 40 days. The ventral side was kept in a phosphate buffer, pH 7.4. Descending and ascending humidity regimens were evaluated. Flux was depicted to be increased from 0.175 g/cm^2^/h (15% relative humidity) to 0.527 g/cm^2^/h (100% relative humidity). A sudden increment in flux (twofold) was observed in the zone of 80–100% relative humidity. This was the humidity zone in which the nail water content enhanced most frequently. Increased relative humidity from 15 to 100% improvised the permeation of radiolabel related to [3H]-ketoconazole three times. Therefore, it was concluded that formulations which cause an increase in nail hydration possessed tremendous potential to improvise topical therapy for the management of onychomycosis [61].

In a report by Hui et al., penetration enhancement effect of 2-n-nonyl-1,3-dioxolane on econazole deep into the human nail was studied. Aliquots (10 microL) of Econail lacquer formulation containing 0.45 mg of [(14)C]-econazole with 18% 2-n-nonyl-1,3-dioxolane (test group) or without 2-n-nonyl-1,3-dioxolane (control group) were applied twice daily for 14 days to human nails that had been washed with ethanol before each morning's application. The hydration of the nail sample was well controlled to simulate normal physiological conditions. After 14 days of dosing, the inner ventral section of the nail plate was assayed for absorbed drug content, using a micrometer-controlled drilling and nails powder removal system. The mass balance values of [(14)C]-econazole in this study were 90.8 and 96.4% for the test and control groups, respectively. The weight-normalized econazole content in the ventral/intermediate nail plate center in the test group was 6 times more than the control (*p* = 0.008). The total econazole absorbed into the supporting bed cotton ball in the test group was nearly 200 times higher than the control group (*p* = 0.008) over the 14-day period. The surface nail contained more econazole, that is, unabsorbed drug, where 2-n-nonyl-1,3-dioxolane was not part of the dosing solution. However, the concentration in the deep nail layer in the test group is 14,000-fold higher than minimum inhibitory concentration believed necessary to inhibit the growth of infecting fungi. In a subsequent study, [(14)C]-dioxolane did not penetrate the nail well. Therefore, the mechanism of penetration enhancement of econazole was found to be at formulation/nail interface [62].

## 7. Clinical Status of Antifungals

Clinical trials of transungual delivery are initiated with the finding that drugs administered topically could enter deep into the nails. Safe and efficacious transungual transport of topical antifungal drugs in onychomycosis has been impeded by less nail permeability. To exert effective action antifungals must acquire therapeutic efficacy and can efficiently undergo permeation across the nail plate [27]. Various clinical trials have been conducted on antifungals and are still in ongoing process to evaluate the potential of antifungals in treating onychomycosis. Some newer antifungals like efinaconazole and oxaboroles like tavaborole and AN2690 have also been investigated for their effectiveness in treating onychomycosis. Oxaboroles were designed with properties believed to be required to allow for easier transit through the nail plate. Hui et al. had reported the nail penetration results of four oxaboroles that led to the selection of AN2690, the results of the nail penetration of AN2690 from four vehicles, and the nail penetration of AN2690 in its chosen vehicle compared to a commercial control, ciclopirox. It was reported by the authors that AN2690 has superior penetration compared to ciclopirox and achieves levels within and under the nail plate that suggest that it has the potential to be an effective topical treatment for onychomycosis [63].  Table 4 lists the clinical status of antifungal drugs used in onychomycosis.

## 8. Patent Reports on Nail Lacquer

While a number of research reports can be found in literature, various patents have also been reported specifying the commercial potential of nail lacquers for transungual drug delivery.  Table 5 highlights several patents on transungual drug delivery via nail lacquers.

## 9. Emerging Topical Therapies for Onychomycosis: Future Perspective

Topical therapy in treating onychomycosis has been found to be not as effective as minimum amount of drug penetrating into the nail plate. Nail morphology specifically its thickness and compact construction makes it a barrier to the entry of topically applied agents. Concentration of the drug across the nail falls about 1000 times from outer to the inner surface. As an outcome, the drug concentration presumably had not reached a therapeutically effective level in the inner ventral layer [8]. The limited success rate of topical therapy in nail disorders is mainly because of the low permeability of keratinized nail plates [64]. The existing clinical reports suggested that a key to successful treatment of onychomycosis by a topical antifungal product lies in overcoming the nail barrier. Recently, available topical treatments via lacquers have limited effectiveness, possibly because they cannot sufficiently penetrate the nail plate to transport a therapeutically sufficient quantity of antifungal drug to the target sites and eradicate the infection. To achieve an effective chemical concentration into/through the human nail plate, penetration enhancers are required to supplement the therapy that tend to promote diffusion through the skin's horny layer [8]. This might be the reason behind the limited effectiveness of nail lacquers. Preparation of nail lacquers also requires the use of penetration enhancers to deliver the drug effectively in the nail bed. As reported by Joshi et al. [7], permeation enhancer thioglycolic acid was incorporated to enhance the penetration. Further, viscosity modification is another issue that has to be looked after. Too low viscosity could pose a threat in its handling, that is, why adjustment of viscosity is necessary so that it can remain on the lacquer brush and can easily flow to cover the nail. Furthermore, to improvise the penetration, a humectant can also be delivered which can modify the structural integrity of nail barrier and provides better results. Panthenol, the alcohol form of pantothenic acid (vitamin B5), is believed to act as a humectant and improve the flexibility and strength of nails as suggested by Hui et al. A liquid nail treatment formulated with panthenol (2%) was compared to a solution of panthenol (2%) in water. Fingernail specimens were dosed daily for 7 days with either the nail treatment (nonlacquer film forming) formulation or aqueous solution with sampling performed every 24 h. Panthenol concentrations were determined in the dorsal surface, interior (by drilling and removal), and the supporting bed under the human nail. Panthenol levels in the dorsal nail, nail interior, and nail supporting bed showed a significant linear increase with each day of dosing. Significantly more panthenol was delivered into the interior nail and supporting bed by a nail treatment formulation than from an aqueous solution. The film not only acts as a reservoir of panthenol but also acts to increase the hydration of the nail and the thermodynamic activity of panthenol as well, thereby enhancing diffusion [65].

However, rising treatment modalities rely prominently on the application of chemicals as well as physical approaches that initiate the persistence and penetration of antifungal agents in infected nail. Physical enhancing method based on iontophoresis could prove to be more fruitful than chemical methods. This methodology has been employed in order to improvise terbinafine delivery into the nails. The cumulative amount of released drug* in vitro* was found to be more than twice above the minimum inhibitory concentration. Thus iontophoresis improved terbinafine delivery into and across the nail plate [66]. Laser therapy that results in formation of partial microsize holes in the nail plate facilitated better terbinafine penetration via application of lacquer solution. This treatment approach is being investigated in Europe for its possible commercialization [67]. Laser therapy is considered as a nonpharmacological approach for treating onychomycosis. Laser systems are endorsed by FDA on the basis of resemblance to established devices and are recommended to temporarily facilitate clear nail in onychomycosis. Neodymium-yttrium garnet lasers classified as short pulse and long pulse lasers and q-switched laser systems have been investigated to increase the mycological cure rates [59].

Mechanical removal of infected nail might be rapidly and easily carried out by a nail clipper. Chemical avulsion of the nail is carried out employing 40% urea, which is applied on the infected nail under occlusion (7 to 14 days). After nail removal, an antimycotic agent like bifonazole is then applied topically up to 4 weeks [68]. A new substitute in the effective management of onychomycosis might be a photodynamic therapy, which was suggested in patients having a contraindication for systemic treatment. After the nail removal, 5-aminolevulinic acid methyl ester (20% solution) was used, along with treatment by radiation of the nail with excimer laser 630 nm at 100 J/cm^2^ (six to seven treatments) until completely curable results were obtained [9]. Photodynamic therapy was also used effectively in another patient suffering with onychomycosis caused by* T. rubrum* that was found to be unresponsive to the previously given treatment. The nail plate was then softened by 40% urea (up to 7 days) under occlusive conditions. The keratotic debris was then removed, and 5-aminolevulinic acid was applied up to 3 h under occlusive conditions. Broadband red light (630 nm) was applied at 37 J/cm^2^ for 7 min 24 sec for removal of dermatophytes, which was then revealed by direct mycologic investigation and culture. Repeated treatment (twice) with an interval of 2 weeks was fixed. No side effects were mentioned. However, complete removal of hyperkeratotic material and infected nail before using photodynamic therapy was vital to get optimum outcomes [69].

In order to treat and manage onychomycosis, topical therapy is supplemented with the systemic therapy to gain more effective results and to cure the disease efficiently. To design optimum dosage regimen various topical products are being used in combination with oral antifungals drugs. Some of these are discussed in the preceding text. Itraconazole in a daily dose of 200 mg daily up to six weeks can be used in combination with amorolfine (5%) nail lacquer used once in a week up to six months. This combination therapy showed up to 84% clinical and mycological curing rate. This finding reached up to 94%, when itraconazole continued to be used for 12 weeks along with nail lacquer (amorolfine) for further six months. When itraconazole was used all alone, the mycological and clinical curable rate was found to be 69% [70]. Another example of combination therapy involves the use of fluconazole (150 mg) once in a week along with amorolfine (5%) nail lacquer once in a week. This combination showed the curing rate in between 75% and 86% [71]. In another investigation conducted on 157 patients treatment was given by applying amorolfine nail lacquer once in a week for 12 months along with an oral antifungal drug (terbinafine) once in a day up to 3 months. Further, itraconazole pulse therapy for 3 months, and fluconazole once in a week for 6 months, depicted same curable rate (71% to 73%) in the three groups [72]. Combined therapy using itraconazole and terbinafine together was compared with terbinafine alone in an investigation on 190 patients up to 72 weeks. Itraconazole pulse used for 1 week in a dose of 400 mg each month for 2 months, followed by one or two additional pulses of terbinafine (500 mg daily for 1 week per month), was then compared with three to four pulses of terbinafine alone. Pulsed sequential therapy provided clinical (56.0% versus 38.9%) and mycologic (72.0% versus 48.9%) curable rates superior to those obtained by the use of terbinafine alone [73]. These findings suggest the effectiveness of combinational therapy employing topical as well as systemic therapy altogether.

## 10. Development of Penetration Assays and Their Comparative Analysis

In order to analyze the penetration of drug deep into the nail bed, several penetration assays have been developed. These assays were designed to investigate amount of drug absorbed in the deeper layers after the application of topical dose. Hui et al. developed an assay procedure to perform comparative analysis of nail drug penetration using penetrating enhancing formulation (test formulation). Test formulation and saline formulations were tested using radiolabeled urea, ketoconazole, and salicylic acid. Inner portions of nail plate were assayed, after twice dosing daily application for 7 days to find out the absorbed drug content via unique drilling/removal system. The findings suggested that the radioactivity contents of three chemicals in the inner intermediate nail plate center in the test formulation were two times higher than the saline formulation. Further, in saline formulation, salicylic acid depicted maximum binding to the outer nail, thus making it less bioavailable for the inner nail area. This was not observed in case of carrier formulation. Thus, it was concluded by the authors that topical treatment in case of onychomycosis is not yet proved to be that much effective, as minimum amount of drug penetrates into the inner nail plate where the disease actually resides. The assay system developed has the unique feature of assaying the inner part of the nail where the disease is perpetuated [74]. Chemical enhancers have been employed to improvise the drug transport across the nail plate. However, selecting an effective chemical enhancer for the given drug and formulation is highly essential in determining the efficacy of topical therapy of nail diseases. Keeping this objective, Murthy et al. have developed TranScreen-N, a high throughput method of optimizing transungual drug permeation enhancers. It is a rapid microwell plate based methodology comprising two different treatment aspects: the simultaneous exposure treatment and the sequential exposure treatment. Various enhancers were screened by TranScreen-N and by diffusion studies employing Franz diffusion cell. In TranScreen-N technique, the enhancers can be grouped according to whether they need to be applied before or simultaneously with drugs (or by either procedure) to enhance the drug delivery across the nail plate. This technique can remarkably minimize the cost and duration required to screen the enhancers [75].

A hydration controlled nail incubation system was also devised in order to monitor the penetration. Nail samples were kept in a Teflon one-chamber diffusion cell in order to maintain the physiological conditions of temperature and humidity. This system was comprised of nail surface that was open in the air and inner surface which was in contact with the cotton ball and placed on the chamber beneath the nail plate. Degree of hydration was observed and determined. In an experiment by Murthy and Maibach, it was observed that average hydration of the wet cotton balls resembled the hydration of the human nail bed obtained from human cadaver. The main benefit of this system was that it provided nonocclusive and hydration controlled environment where normal physiological conditions can be achieved. However, to analyze the drug concentration drilling system was developed which is comprised of a nail sampling stage and a drill, which provided the possibility to collect the sample from inner region of the nail [76]. Thus, development of these methodologies provides an easy access to evaluate the penetrability across the nail bed.

## 11. Concluding Remarks

Limited permeability of antifungals across the nail poses a threat in transungual delivery in order to treat deep seated nail fungal infections. Nail lacquers have been suggested to possess efficient permeability across the nail plate. A diverse range of antifungals like azoles and allylamines have been investigated for the management of onychomycosis. Delivery of these drugs via nail lacquers is found to be safe and effective in the therapy for onychomycosis.

## Figures and Tables

**Table 1:** A compilation of commercially available nail lacquers.

Therapeutic agent	Brand name	Company name
Ciclopiroxamine 8%	Onlyac®	Dermic, Canada
Ciclopiroxamine 8%	Penlac®	Roche lab, Australia
Ciclopiroxamine 8%	Nailon®	Protech Biosystem, India
Amorolfine 5%	Loceryl®	Protech Biosystem, India
Econazole 5%	Econail®	Macrochem Corporation, Lexington [12]

**Table 2:** Antifungal agents utilized in the treatment of onychomycosis.

Therapeutic category	Examples of antifungals	Organisms	Reference
Allylamines and benzylamines	Terbinafine-HCl, naftifine, butenafine	*T. rubrum*, *T. mentagrophytes*, *T. tonsurans*, *E. floccosum* (fungicidal effect) *Candida *spp., *Scopulariopsis *spp., *Aspergillus *spp. (fungi- static activity)	[13]
Azoles	Itraconazole, fluconazole, posaconazole, voriconazole, ravuconazole, isavuconazole, pramiconazole, albaconazole, efinaconazole	*C. tropicalis*, *C. glabrata*, *C. krusei*, *Zygomycetes*, *Cryptococcus neoformans*	

**Table 3:** Topical drug delivery via several other carriers for the treatment of onychomycosis.

Drug	System	Concluding remarks	Reference
Ciclopirox	Gel	Enhanced permeation into the nail	[13]
Terbinafine	Transfersomes	Significant antifungal activity	[14]
Itraconazole	Microemulsion gel	Microemulsion based gel exhibited better retention, penetration, and antifungal activity	[15]
Fluconazole	Microemulsion gel	Better antifungal activity against *Aspergillus niger* as compared to the commercial gel	[16]
Ciclopirox	Nanoemulsion gel	Better retention ability was observed for nanoemulsion based gel	[17]
Terbinafine-HCl	Liposomes/ethosomes	Effective formulations for ungual delivery, suggesting that liposome based poloxamer gel formulation could be a potential carrier for ungual drug delivery	[18]
Ciclopirox	Formulation based on lipid diffusion enhancers	Effective in the topical treatment of onychomycosis	[19]
Clotrimazole	Nanoemulsion gel	Effective antifungal activity against *Candida albicans*	[20]
Ciclopirox	Poloxamer 407-based formulations	Permeation coefficient from P407-based formulations was higher in comparison to the nail lacquer	[21]
Efinaconazole	Solution	Effective transungual delivery of efinaconazole	[22]
Terbinafine-HCl	Liposomal film	Liposomal film based formulation showed better antifungal activity on fungal infected nails	[23]
Sertaconazole	Nail penetration enhancer containing nanovesicles (nPEVs)	1.4-fold higher hydration and drug uptake enhancement into nail clippings showed significantly higher zone of inhibition for *Trichophyton rubrum* than the marketed cream	[24]

**Table 4:** Clinical status of antifungal drugs used in onychomycosis.

Therapeutic agent	Clinical status	Reference
Amorolfine/RV4104A/Ciclopiroxolamine	Unknown	[25]
Econail*™* (econazole)	Phase II (completed)	[26]
Phytonail/Loceryl	Unknown	[25]
Terbinafine hydrochloride/amorolfine	Completed	[25]
Terbinafine hydrochloride	Completed	[25]
Ciclopirox	Completed	[25]
Amorolfine/terbinafine	Completed	[25]
MycoVa*™*	Phase III (completed)	[26]
Er:YAG laser (device) with amorolfine	Unknown	[25]
Luliconazole (solution)	Phase IIb/III (ongoing)	[25]
Efinaconazole (10% topical solution)	Phase III (completed)	[27]
TDT067 (liquid spray)	Phase III (ongoing)	[26]
P-3058	Phase II (ongoing)	[26]
Isavuconazole	Phase III (completed)	[28]
Tavaborole	Phase III (completed)	[29]
Albaconazole	Phase II (completed)	[30]
Posaconazole	Phase II (completed)	[30]
VT-1161	Phase II (ongoing)	[31]

**Table 5:** Patent reports on nail lacquer for transungual drug delivery.

Patent/application number (year of issue/publication)	Original assignee/applicant	Comment	Reference
CA2400178 A1 (2001)	Gyurik RJ, Bentley Pharmaceuticals, Inc., Exeter, New Hampshire, USA; Cpex Pharmaceuticals, Inc., Wilmington, Delaware, USA	Depicting an innovation based on a pharmaceutical composition containing a permeation enhancer that enhances the rate of passage of a pharmaceutical compound across the nail or membrane	[32]
US6254878 B1 (2001)	E.I. Du Pont De Nemours & Company, Wilmington, Delaware, USA	Inventing acrylic based nail polish and its composition containing binders and dispersants or both	[33]
US6391879 B1 (2001)	Astan, Inc., Birmingham, Alabama, USA	Disclosing a therapy and its application in the treatment of nail fungal infection (onychomycosis) composed of an antifungal drug with DMSO in polyglycolic solution	[34]
US6495124B1 (2002)	Macrochem Corporation, Wellesley, Massachusetts	Describing nail lacquer for fungal infections containing drugs like econazole/ciclopirox in a polymeric film of pentadecalactone/lactone and a solvent (volatile)	[35]
US6622064 B2 (2003)	Imx Labs, Inc., Birmingham, Oakland, Michigan	Disclosing a nail polish color. Its methods and dispensing have been described	[36]
US8771655 B2 (2014)	Debra Marino	Revealing a naturally developed nail polish prepared with fruits/vegetables containing acrylic polymer	[37]
US20120178056A1 (2012)	Cindy Nelson	Disclosing a colored and translucent nail polish prepared with different colors	[38]
US20130149266 A1 (2013)	Michael Mitsuo Homma, Victor Masaru Homma	Providing long residing and easily dried nail polish comprising a lacquer and pigment composition	[39]
US20150190331 A1 (2015)	Pakaly Enterprise Co., Ltd., Chung-Ho, Taipei, Taiwan	Inventing a gel based polish comprising (meth)acrylate monomer like ethyl methacrylate and hydroxyethylmethacrylate	[40]
WO2003007675 A2 (2003)	Benny Borsakian, Janel Faraci	Disclosing a nail polish preparation containing a base, colorant, and a photochromic powder	[41]
